# Clinico-radiological features of intracranial atherosclerosis-related large vessel occlusion prior to endovascular treatment

**DOI:** 10.1038/s41598-024-53354-z

**Published:** 2024-02-05

**Authors:** Marc Rodrigo-Gisbert, Alvaro García-Tornel, Manuel Requena, Isabel Vielba-Gómez, Saima Bashir, Marta Rubiera, Marta De Dios Lascuevas, Marta Olivé-Gadea, Carlos Piñana, Federica Rizzo, Marian Muchada, Noelia Rodriguez-Villatoro, David Rodríguez-Luna, Jesus Juega, Jorge Pagola, David Hernández, Carlos A. Molina, Mikel Terceño, Alejandro Tomasello, Marc Ribo

**Affiliations:** 1 Stroke Unit, Department of Neurology, Hospital Universitari Vall d’Hebron, Passeig de La Vall d’Hebron 119-129, 08035 Barcelona, Spain; 2 Departament de Medicina, Universitat Autònoma de Barcelona, Barcelona, Spain; 3 Department of Neuroradiology, Hospital Universitari Vall d’Hebron, Barcelona, Spain; 4 Stroke Unit, Department of Neurology, Hospital Universitari Dr. Josep Trueta, Girona, Spain

**Keywords:** Neuroscience, Neurology, Stroke

## Abstract

The identification of large vessel occlusion with underlying intracranial atherosclerotic disease (ICAS-LVO) before endovascular treatment (EVT) continues to be a challenge. We aimed to analyze baseline clinical-radiological features associated with ICAS-LVO that could lead to a prompt identification. We performed a retrospective cross-sectional study of consecutive patients with stroke treated with EVT from January 2020 to April 2022. We included anterior LVO involving intracranial internal carotid artery and middle cerebral artery. We analyzed baseline clinical and radiological variables associated with ICAS-LVO and evaluated the diagnostic value of a multivariate logistic regression model to identify ICAS-LVO before EVT. ICAS-LVO was defined as presence of angiographic residual stenosis or a trend to re-occlusion during EVT procedure. A total of 338 patients were included in the study. Of them, 28 patients (8.3%) presented with ICAS-LVO. After adjusting for confounders, absence of atrial fibrillation (OR 9.33, 95% CI 1.11–78.42; *p* = 0.040), lower hypoperfusion intensity ratio (HIR [Tmax > 10 s/Tmax > 6 s ratio], (OR 0.69, 95% CI 0.50–0.95; *p* = 0.025), symptomatic intracranial artery calcification (IAC, OR .15, 95% CI 1.64–26.42, *p* = 0.006), a more proximal occlusion (ICA, MCA-M1: OR 4.00, 95% CI 1.23–13.03; *p* = 0.021), and smoking (OR 2.91, 95% CI 1.08–7.90; *p* = 0.035) were associated with ICAS-LVO. The clinico-radiological model showed an overall well capability to identify ICAS-LVO (AUC = 0.88, 95% CI 0.83–0.94; *p* < 0.001). In conclusion, a combination of clinical and radiological features available before EVT can help to identify an ICAS-LVO. This approach could be useful to perform a rapid assessment of underlying etiology and suggest specific pathophysiology-based measures. Prospective studies are needed to validate these findings in other populations.

## Introduction

In the era of endovascular treatment (EVT) for acute ischemic stroke with large vessel occlusion (LVO)^1,2^, the optimal EVT strategy for the treatment of underlying intracranial atherosclerotic disease (ICAD) remains unclear.

Mechanical thrombectomy (MT) in patients with underlying ICAS-LVO can be challenging and remains the most common cause of failed reperfusion using usual MT strategies. In these cases, MT has been associated with longer procedural time with higher complications rate, and worse angiographic and clinical outcomes^3^. Although there is a paucity of randomized data, rescue MT with balloon angioplasty and/or stenting is often necessary and has proven to be technically feasible and effective^4^.

The diagnosis of ICAS-LVO prior to EVT could be useful to optimize patient selection and perform a patient-tailored thrombectomy anticipating rescue treatments such as mechanical or pharmacological interventions. However, the diagnostic performance of clinical and radiological biomarkers to detect underlying ICAD before EVT has not been systematically studied. Previous studies suggest that automated parameters of computed tomography perfusion (CTP) associated with a good collateral status are associated with underlying ICAD^5–9^.

For this single center study, we aimed to evaluate a combination of clinical and radiological features available before EVT to help to quickly identify the presence of ICAS-LVO.

## Methods

This study followed the Strengthening the Reporting of Observational Studies in Epidemiology (STROBE) statements, all procedures were conducted in strict adherence to applicable guidelines and regulations^10,11^. The study protocol was reviewed and approved by Vall d’Hebron Hospital Ethics Committee, approval number (PR(AG)434/2023). Because of the retrospective nature of this study, the need for written informed consent was waived by the Vall d’Hebron Hospital Ethics Committee.

### Data availability

Anonymized data supporting the current study’s findings are available for any qualified investigator upon reasonable request to the corresponding author.

### Study design and population

**Figure 1:**
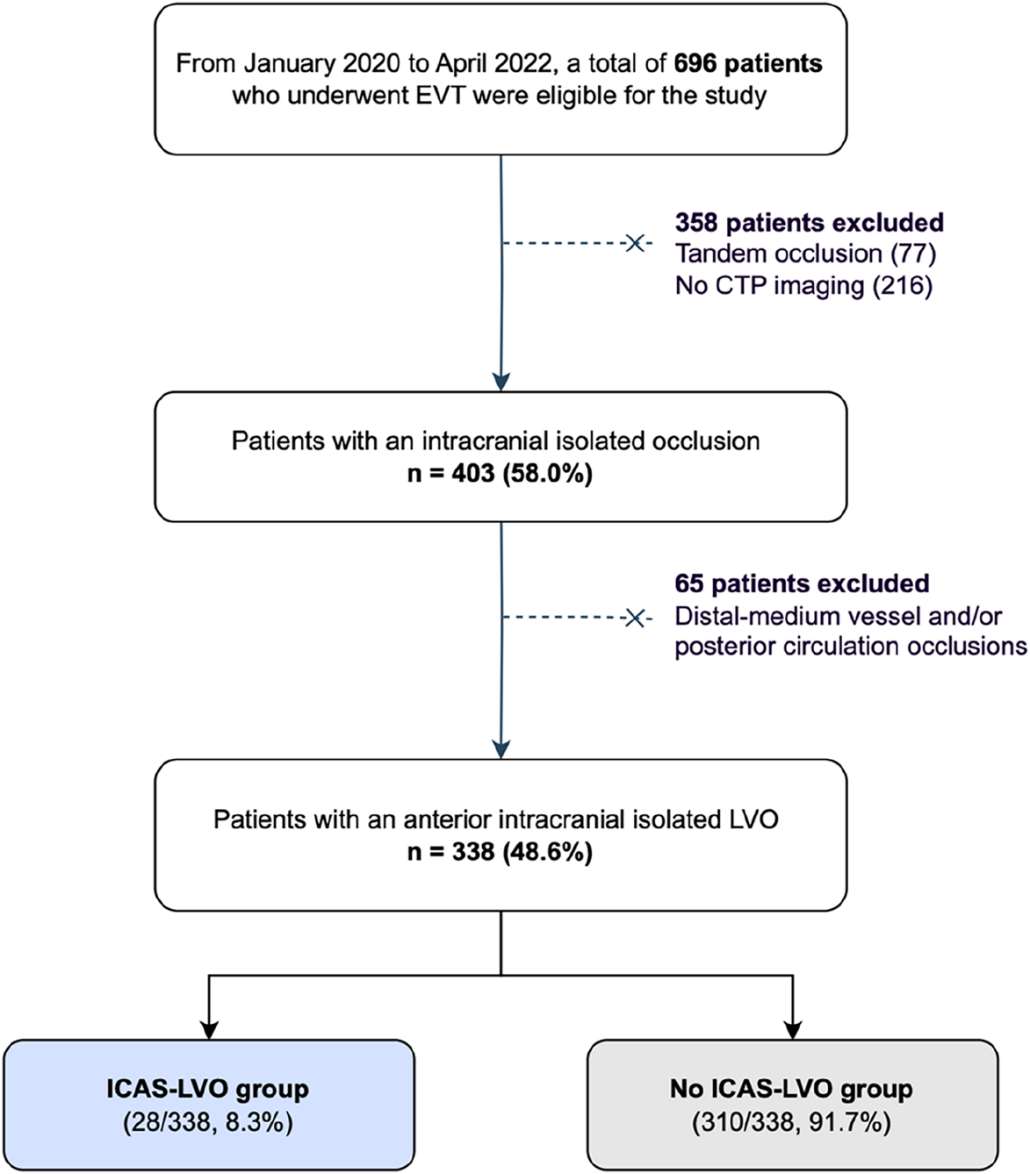
Patient selection flow chart. Footnote: EVT, endovascular treatment; CTP, computed tomography perfusion; LVO, large vessel occlusion; ICAS-LVO, intracranial atherosclerosis related large vessel occlusion.

We performed a cross-sectional study based on a prospectively maintained single-center database of patients with an acute ischemic stroke undergoing reperfusion treatments. We included patients with an anterior intracranial LVO (intracranial internal carotid, middle cerebral artery segments M1 and M2) who had baseline non-contrast CT, CT angiography and CT perfusion and underwent EVT from January 2020 to April 2022. Exclusion criteria were admission after 24 h from stroke symptoms onset and the presence of an extracranial occlusion of the internal carotid artery. A flow chart of the included patients and reasons to exclusions is represented in Fig. 1.

### Clinical and radiological parameters

Recorded demographic and clinical variables included age, sex, baseline modified Rankin Scale (mRS), medical comorbidities, stroke severity assessed by National Institutes of Health Stroke Scale (NIHSS), admission Alberta Stroke Program Early CT score (ASPECTS), workflow times (symptom onset, imaging, and groin puncture), and reperfusion therapies administered. The degree of reperfusion was determined prospectively by consensus between the interventionalist and the vascular neurologist immediately after the procedure using the expanded Thrombolysis in Cerebral Ischemia (eTICI) score. Patients were considered to achieve successful recanalization if at least 50% of downstream reperfusion was achieved (eTICI ≥ 2B).

A symptomatic intracranial artery calcification (IAC) was defined as the presence of IAC with intimal pattern in the occlusion location or immediately proximal, in direct contact with the occlusion, as previously described^12^. Calcifications were registered on admission non-contrast CT with a slice thickness ≤ 1 mm and were considered for values > 130 HU, analogous to cardiology scoring systems^13^. IAC pattern in LVO was differentiated into intimal and medial IAC according to a previously validated score, which assesses thickness (1 point for ≥ 1.5 mm, 3 for < 1.5 mm), circularity (1 point for dot, 2 for < 90°, 3 for 90°–270°, and 4 for 270°–360°), and morphology along the long axis of the artery (1 for irregular/patchy, 4 for continuous, and 0 points for indistinguishable). Indistinguishable was used to describe dot like calcifications that were too small to assign any morphological characteristics to, and were therefore not categorizable. The score designates an intimal IAC when ranged from 1 to 6 points, and a medial IAC when ranged from 7 to 11 points^14^.

Automated CTP parameters were assessed by RAPID® software (iSchemaView, Menlo Park, CA) on baseline neuroimaging study. We included cerebral blood flow (CBF) < 30%, T_max_ > 4 s, T_max_ > 6 s, T_max_ > 10 s, mismatch volume (difference between T_max_ > 6 s and CBF < 30%), hypoperfusion intensity ratio (T_max_ > 10 s/T_max_ > 6 s ratio, HIR), and T_max_ > 4 s/T_max_ > 6 s ratio. HIR was used to determine collateral status as favorable (HIR < 0.25) and unfavorable (HIR > 0.25) based on ROC analysis and previously published thresholds^7,15^.

We defined ICAS-LVO by angiographic residual fixed stenosis > 50% after successful reperfusion in the target vessel with distal flow impairment or a trend to re-occlusion during the EVT procedure. The residual stenosis degree was assessed according to the Warfarin Aspirin Symptomatic Intracranial Disease criteria^16^. Re-occlusion was defined as an immediate re-occlusion of the target vessel during EVT procedure after initial revascularization. Stroke mechanism work-up included heart rhythm monitoring for at least 72 h, imaging of both the extracranial and intracranial arteries supplying the area of brain ischemia with CT angiography, precordial echocardiography, and a comprehensive evaluation if rare causes were suspected.

### Statistical analysis

Kolmogorov–Smirnov and Shapiro–Wilk tests were used to assure normality of continuous variables. Categorical variables were presented as absolute values and percentages and continuous variables as median (interquartile range (IQR)) or means (± standard deviation (SD)) as indicated. Statistical significance for intergroup differences was assessed by Pearson χ2 test or Fisher exact test for categorical variables and by Mann–Whitney U test or Student t-test as appropriate to continuous variables.

A multivariable binary regression model was constructed using the Least Absolute Shrinkage and Selection Operator (LASSO) method to identify a subset of variables independently associated with underlying ICAD. The LASSO method included variables that demonstrated either a statistically significant association or clinical relevance to the explored outcome. The cutoff of HIR with best sensitivity and specificity to predict underlying ICAD was determined through a receiver operating characteristic (ROC) curve, utilizing the Youden's Index. Additionally, a sensitivity analysis was conducted, focusing on patients with successful reperfusion at any time during EVT.

ROC curves were constructed to assess model diagnostic accuracy to detect the presence of underlying ICAD, including independent predictors from the multivariable analysis the following models: (1) radiological variables only, (2) clinical and radiological variable. The diagnostic accuracy of each model was determined based on the area under the ROC curve with a confidence interval (CI) of 95%. Additionally, a comparison between the AUCs of both models was conducted using the DeLong test implemented in R with the *pROC* package.

A *p* value < 0.05 was considered statistically significant. All analyses were performed using the IBM SPSS Statistics (version 25) software and R (version 4.3.0, R Foundation for Statistical Computing, Vienna, Austria).

### Ethical approval

Dr Molina reported receiving personal fees from AstraZeneca for consultant services outside the submitted work. Dr Tomasello reported receiving personal fees from Anaconda Biomed, Balt, Medtronic, Perflow, and Stryker outside the submitted work. Dr Ribo reported receiving personal fees from Anaconda Biomed, AptaTargets, Cerenovus, Medtronic, Methinks, Philips, Sanofi, Stryker, Balt, and Rapid AI outside the submitted work; he has a modest ownership of NoraHealth. The other authors report no conflicts. The authors declare that the research was conducted in the absence of any commercial or financial relationships that could be construed as a potential conflict of interest.

## Results

### Baseline characteristics and univariate analysis

From January 2020 to April 2022, a total of 695 patients with an acute ischemic stroke that underwent EVT were eligible for the study. We included 338 patients (48.6%) who met the eligibility criteria, of whom ICAS-LVO was diagnosed in 28 patients (8.3%). The median age was 79 (IQR 66–86) years, 196 patients (58.0%) were women, and the median baseline mRS was 1 (IQR 0–2). In our population, only a single patient of Asian ethnicity was included in the non-ICAD group (1/338, 0.3%).

Patients with ICAS-LVO were younger (70 years [IQR 66–86] vs 80 years [IQR 67–86]), had a higher proportion of former/current history of smoking (57.1% [16/28] vs 23.1% [72/310]), a lower proportion of atrial fibrillation diagnosis (3.6% [1/28] vs 35.5% [110/310]), and a lower median admission NIHSS score (11 [IQR 8–18] vs 16 [IQR 10–20]). CTP-derived perfusion maps showed that patients with ICAS-LVO had a smaller ischemic core volume (median 0 mL [IQR 0–7] vs 6 mL [IQR 0–26]), a higher proportion of T_max_ > 4 s/T_max_ > 6 s ratio > 2 (53.6% [15/28] vs 33.9% [105/310]), and a lower hypoperfusion intensity ratio (HIR) (median 0.16 [IQR 0–0.37] vs 0.44 [IQR 0.29–0.57]). No significant differences were observed in other baseline characteristics. Table 1 summarizes the baseline characteristics and demographics according to the diagnosis of ICAS-LVO.

**Table 1:** Baseline characteristics and demographics.

	All patients (n = 338)	ICAS-LVO (n = 28)	No ICAS-LVO (n = 310)	*p* Value
Age (years) [median, IQR]	79 (66–86)	70 (62–77)	80 (67–86)	< 0.001
Sex (female) [n, %]	196 (58.0%)	16 (57.1%)	180 (58.1%)	0.925
*Risk factors* [n, %] Former or current smoker Hypertension Diabetes mellitus Dyslipidemia Atrial fibrillation Ischemic heart disease Active oncological disease Previous stroke	88 (26%) 230 (68.0%) 92 (27.2%) 167 (49.4%) 111 (32.8%) 59 (17.5%) 16 (4.7%) 69 (20.4%)	16 (57.1%) 18 (64.3%) 8 (28.6%) 13 (46.4%) 1 (3.6%) 3 (10.7%) 0 (0%) 6 (21.4%)	72 (23.3%) 212 (68.4%) 84 (27.1%) 154 (49.7%) 110 (35.5%) 56 (18.1%) 16 (5.2%) 63 (20.3%)	< 0.001 0.656 0.867 0.742 0.001 0.323 0.678 0.889
Premorbid mRS [median, IQR]	1 (1–2)	1 (0–2)	1 (1–2)	0.413
Baseline NIHSS [median, IQR]	16 (10–20)	11 (8–18)	16 (10–20)	0.024
*Occlusion level* [n, %] Intracranial ICA MCA M1 MCA M2	65 (19.2%) 152 (46.2%) 117 (34.6%)	7 (25%) 15 (53.6%) 6 (21.4%)	58 (18.7%) 41 (45.5%) 111 (35.8%)	0.297
Proximal occlusion (Intracranial ICA plus MCA M1) [n, %]	223 (66.0%)	22 (81.5%)	201 (64.6%)	0.076
Wake-up stroke [n, %]	129 (38.2%)	14 (50%)	115 (37.1%)	0.178
Onset to imaging [mean ± SD]	323 ± 287	412 ± 292	315 ± 285	0.098
Onset to groin time [mean ± SD]	393 ± 296	452 ± 299	388 ± 296	0.265
ASPECTS [median, IQR] [n, %]	9 (8–10)	9 (8–10)	9 (8–10)	0.256
Symptomatic IAC [n, %]	19 (5.6%)	6 (21.4%)	13 (4.2%)	0.002
CTP automated parameters				
CBF < 30%	5 (0–24)	0 (0–7)	6 (0–26)	0.004
Mismatch volume	78 (52–116)	75 (37–128)	78 (52–115)	0.078
Tmax > 4 s/Tmax > 6 s	1.78 (1.54–2.27)	2.01 (1.59–2.61)	1.77 (1.52–2.24)	0.909
Tmax > 4 s/Tmax > 6 s > 2	120 (35.5%)	15 (53.6%)	105 (33.9%)	0.037
HIR	0.43 (0.26–0.57)	0.16 (0–0.37)	0.44 (0.29–0.57)	< 0.001
HIR < 0.25	79 (23.4%)	18 (66.7%)	61 (19.7%)	< 0.001

ICAD, intracranial atherosclerotic disease; mRS, modified Rankin scale; NIHSS, National Institutes of Health Stroke Scale; ICA, internal carotid artery; MCA, middle cerebral artery; ASPECTS, Alberta Stroke Program Early CT score; IAC, intracranial artery calcification; CBF, cerebral blood flow; HIR, hypoperfusion intensity ratio.

### Multivariable and ROC analysis

**Figure 2:**
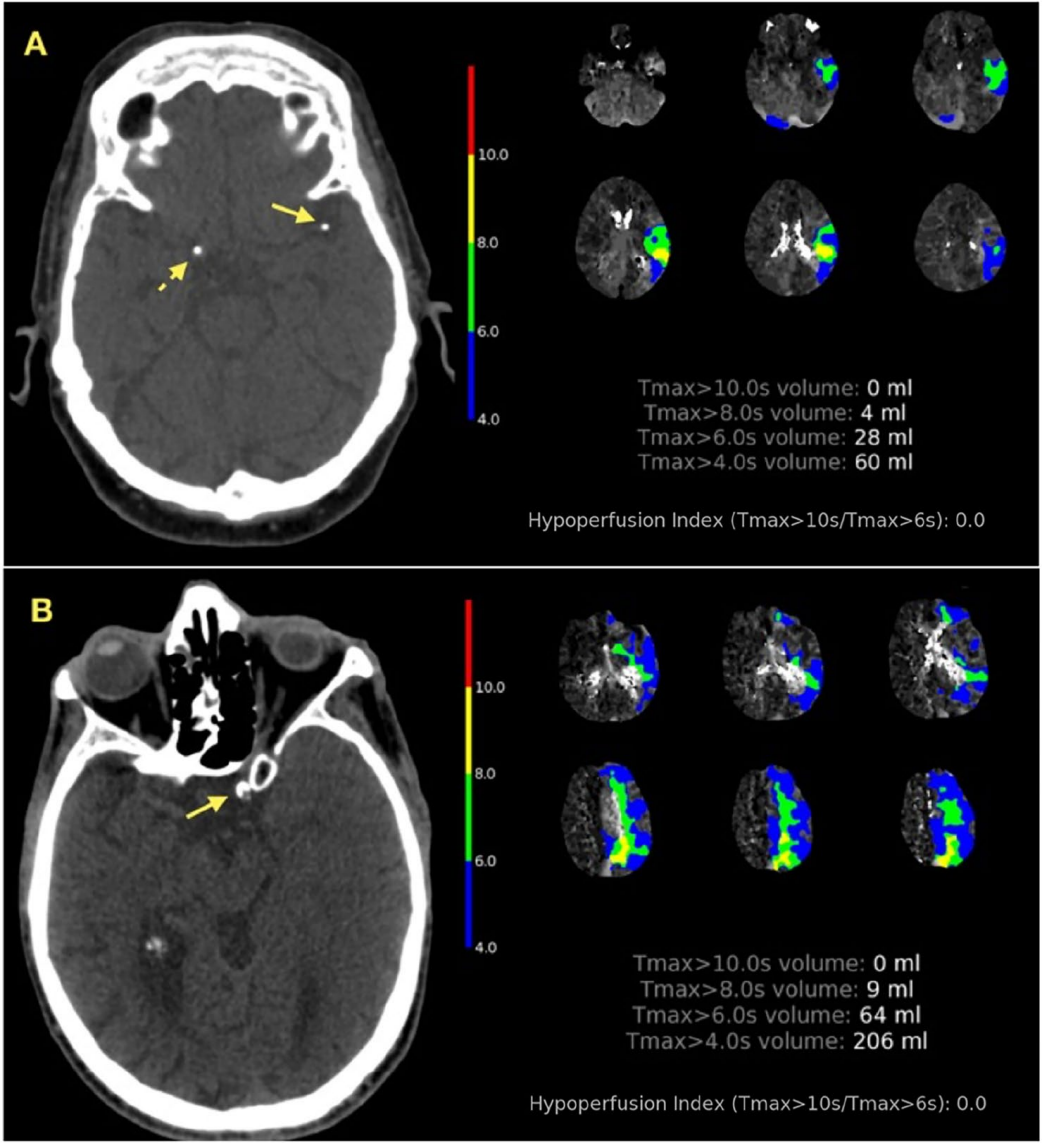
Illustrative cases demonstrating intracranial atherosclerosis-related large vessel occlusions biomarkers. (**A**) Patient with left MCA segment M1 occlusion; and (**B**) patient with left intracranial ICA occlusion. In both cases, axial non-contrast CT with symptomatic intracranial artery calcification in occlusion location (yellow arrow), asymptomatic IAC (dotted arrow, only patient **A**); and CTP automated parameters including HIR < 0.25.

After adjusting for identifiable baseline confounders such as age, baseline NIHSS score, T_max_ > 4 s/T_max_ > 6 s > 2, and ischemic core (CBF < 30%), the absence of atrial fibrillation (OR 9.33, 95% CI 1.11–78.42; *p* = 0.040), the presence of symptomatic IAC (OR 0.15, 95% CI 1.64–26.42, *p* = 0.006), lower HIR (OR 0.69, 95% CI 0.50–0.95; *p* = 0.025), a proximal occlusion (ICA, MCA-M1: OR 4.00, 95% CI 1.23–13.03; *p* = 0.021) and smoking habit history (OR 2.91, 95% CI 1.08–7.90; *p* = 0.035) were associated with ICAS-LVO (Table 2 and Fig. 2). On CTP imaging, the best cutoff point to identify ICAS-LVO was HIR < 0.25 according to ROC analyses.

**Table 2:** Binary logistic regression model for detecting large vessel occlusion with underlying ICAD.

	Univariate analysis			Multivariate analysis			
	Unadjusted OR	95% CI	*p* Value	LASSO coefficient	OR	95% CI	*p* value
Absence of atrial fibrillation	14.23	1.91–106.28	0.010	2.29	9.89	1.18–82.97	0.035
Proximal occlusion (intracranial ICA, MCA M1)	2.41	0.89–6.54	0.085	1.42	4.12	1.23–13.38	0.018
Former/current smoker	4.08	1.83–9.10	0.001	1.22	3.38	1.28–8.94	0.014
Symptomatic IAC	6.55	2.26–18.98	0.001	1.95	6.99	1.74–28.05	0.006
HIR (0.1 unit increment)	0.63	0.51–0.78	< 0.001	-0.40	0.67	0.53–0.85	0.001
Age (1 unit increment)	0.96	0.94–0.99	0.004	-0.01	0.99	0.96–1.03	0.686
Baseline NIHSS (1 unit increment)	0.93	0.87–0.99	0.022	-0.04	0.96	0.88–1.05	0.407

ICA, internal carotid artery; MCA-M1, middle cerebral artery segment M1; IAC, intracranial artery calcification; HIR, hypoperfusion intensity ratio; NIHSS, National Institutes of Health Stroke Scale; CBF, cerebral blood flow.

**Figure 3:**
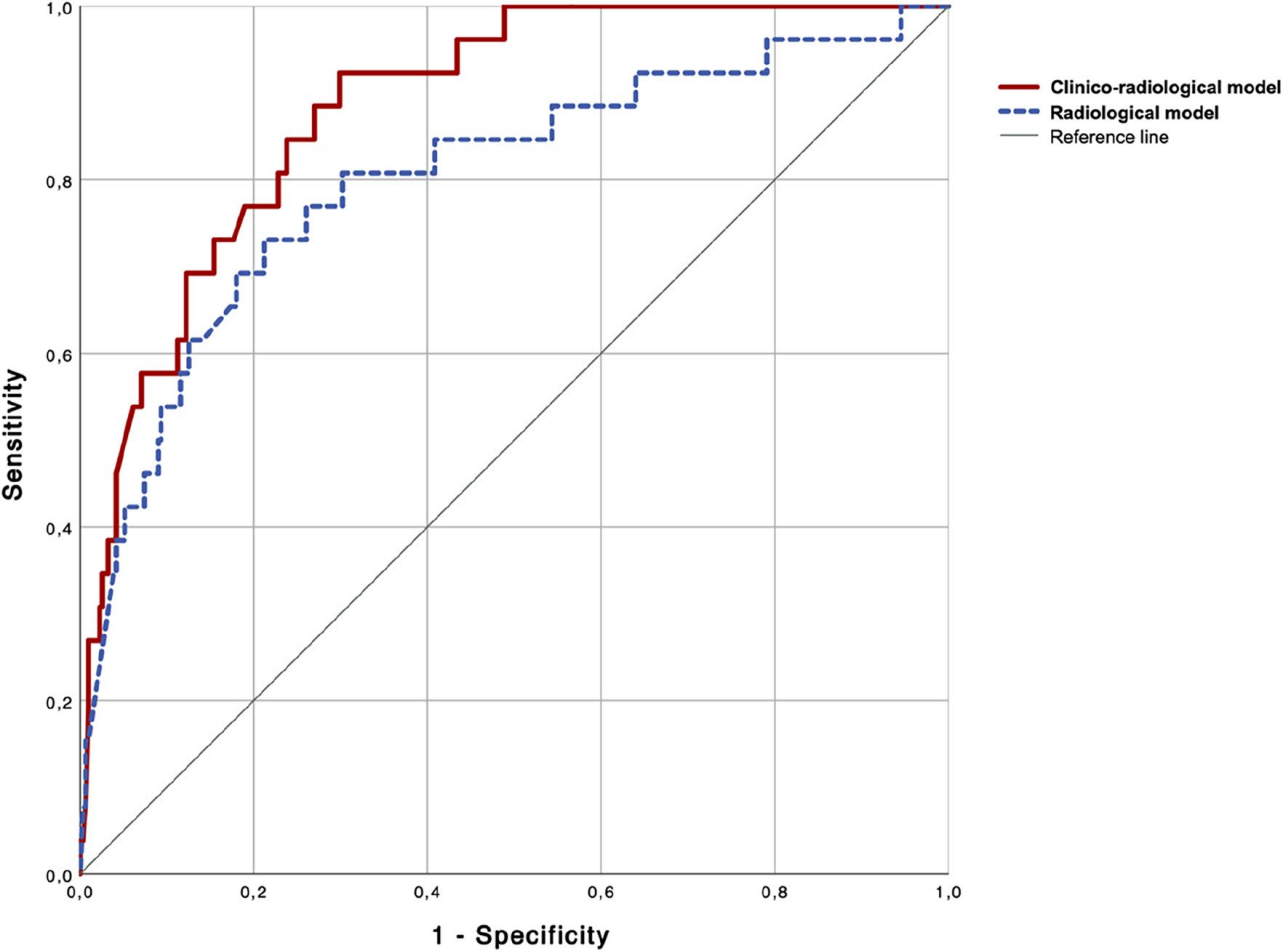
Capability to identify large vessel occlusion with underlying ICAD according to receiver operating characteristic (ROC) analysis. ROC analysis of regression models that include independent predictors of LVO with underlying ICAD in an isolated radiological model including IAC, HIR, and occlusion location (AUC 0.80, 95% CI 0.70–0.90: *p* < 0.001) and a comprehensive clinico-radiological model including smoking and atrial fibrillation in addition to previous radiological features (AUC 0.88, 95% CI 0.83–0.94; *p* < 0.001). Footnote: AUC, area under the curve; IAC, intracranial artery calcifications; HIR, Hypoperfusion intensity ratio.

The probabilities obtained from a multivariate model which includes variables significantly associated with ICAS-LVO showed an overall well capability to identify a LVO with underlying ICAD. The multivariate model with radiological variables, which includes symptomatic IAC, HIR, and occlusion location (AUC 0.80, 95% CI 0.70–0.90; *p* < 0.001), as well as the model which included clinical variables (history of smoking and atrial fibrillation) were added to the radiological variables (AUC 0.88, 95% CI 0.83–0.94; *p* < 0.001). The clinico-radiological model significantly improved the predictive capability to identify an ICAS-LVO compared to the radiological model alone (DeLong test: Z = 2.0116, *p* = 0.044). The ROC curves for both evaluations (radiological and comprehensive clinico-radiological models) are represented in Fig. 3.

### Sensitivity analysis of patients with successful reperfusion

After excluding patients who did not achieve successful reperfusion at any time during the EVT procedure, we analyze a total of 310 patients. Of them, 25 patients (8.1%) were diagnosed with ICAS-LVO. Lower HIR (0.1-unit increment, OR 0.68, 95% CI 0.53–0.87; *p* = 0.002), a proximal occlusion (OR 5.05, 95% CI 1.36–18.75; *p* = 0.016), presence of symptomatic IAC (OR 5.15, 95% CI 1.12–23.65; *p* = 0.035), smoking history (OR 4.04, 95% CI 1.45–11.29, *p* = 0.008), and absence of atrial fibrillation (OR 9.77, 95% CI 1.17–81.89, *p* = 0.036) persisted as independently associated factors with pre-EVT identification of ICAS-LVO.

### Angiographic outcomes

In our cohort, those with an ICAS-LVO exhibited a lower rate of successful reperfusion before the use of rescue treatments (which are defined as intracranial angioplasty + /− intracranial stenting) (eTICI ≥ 2b 21.4% [6/28] vs 89.7% [278/310], *p* < 0.001), a higher rate of rescue treatments (60.7% [17/38] vs 1.6% [5/310], *p* < 0.001) and a lower rate of final successful reperfusion (eTICI ≥ 2b 71.4% [20/38] vs 91.3% [283/310], *p* = 0.003). Complete angiographic outcomes according to the presence of ICAS-LVO are presented in Table S1.

## Discussion

Our study provides evidence supporting the hypothesis that the underlying presence of ICAS-LVO can be accurately predicted before EVT using clinical and radiological variables. Specifically, the presence of intracranial artery calcifications, collateral status on admission CTP, occlusion location, and the presence of atrial fibrillation or smoking habit were significantly associated with ICAS-LVO in our cohort of stroke patients treated with thrombectomy. These findings can orient towards an early identification and management of an underlying atherosclerotic lesion, potentially leading to a tailored EVT procedure and improved clinical outcomes.

The potential benefit of EVT is limited by recanalization failure in a portion of stroke patients that ranges 15–30%^17^. Several factors may contribute to recanalization failure, including clot characteristics such as fibrin-rich or calcified clots, anatomical challenges, and atherosclerotic occlusions^12,18,19^. Currently, the diagnosis of ICAD-related occlusion based on The Trial of Org 10172 in Acute Stroke Treatment (TOAST) classification requires a full diagnosis work-up, delaying the diagnosis after EVT is performed^20^. Unfortunately, there is no standardized criteria for diagnosing underlying ICAD in acute LVO before EVT^21^. To facilitate a timely detection of the condition, current approaches include applying artificial intelligence technology to analyze CTP maps parameters and/or using information related to recanalization status after the first pass during EVT^22,23^.

As showed in our cohort, EVT in ICAS-LVO has been associated with a higher risk of reperfusion failure and remains a therapeutic challenge. Emergent rescue treatments, such as antiplatelet drug adding, angioplasty and/or stent deployment, are often necessary in these cases^4^. A recent cohort analysis with data from the ANGEL-ACT registry has shown that angioplasty and/or stenting is effective and safe in patients with an ICAS-LVO and should be performed at an early stage of the endovascular procedure to increase the chances of success^24^. A diagnostic tool with this clinico-radiological set can optimize patient assessment in order to adopt pathophysiology-based decisions. Implementing upfront the recommended pharmacological and mechanical strategies for ICAD lesions, will avoid extensive activation of the underlying plaque secondary to multiple failed passes with non-specific devices. The EVT strategy often includes an initial loading of antiplatelet drugs and gentle debulking of the lesion with a first thrombectomy attempt, followed by an intracranial angioplasty and eventual stent deployment. Therefore, considering this approach before initiating the procedure or after the first thrombectomy attempt could potentially enhance the detection of those occlusions with a higher odd of no reperfusion, and may also prevent therapeutic inertia using rescue treatments at an early stage.

Multiple studies have analyzed clinical and radiological variables associated with ICAS-LVO^5–7,25^. However, this study combines NNCT findings with CTP-derived parameters and clinical variables to allow an accurate prediction of patients with ICAS-LVO.

In our study, a symptomatic IAC was identified in 21.1% of patients with ICAS-LVO, whereas it was found in only 4.2% of patients with embolic occlusions. This finding has been confirmed in other cohorts of patients with ICAS-LVO, where it is reported in 33% of cases^26^. Hence, symptomatic IAC are associated with unsuccessful reperfusion and could be used as an adjunct biomarker in the diagnosis of intracranial atherosclerotic disease in addition to other imaging features such as the presence intracranial atherosclerotic plaque on MRI or perfusion imaging maps patterns^12^. A preceding study showed that individuals with symptomatic IAC occlusion who underwent rescue procedures (intracranial angioplasty and/or stenting bailout) exhibited similar recanalization rate to that of individuals with non-calcified thrombus^12^. Hence, an early adoption of rescue procedures might lead to improved clinical and angiographic outcomes in patients with an ICAS-LVO in conjunction with IAC.

The hypoperfusion intensity ratio is a reliable indicator of collateral status in acute ischemic stroke, which can be quantitatively and automatically derived from CT perfusion datasets. Although a definitive threshold for defining poor collateral status based on HIR is not well established and may vary between different software programs, Guenego et al. showed that a threshold of HIR < 0.4 was associated with good collaterals on digital substraction angiography^27,28^. A more stringent HIR threshold of < 0.3 has been suggested for excellent collateral status and < 0.22 for predicting underlying ICAD in the anterior circulation LVO^7,15^. Therefore, defining a stricter HIR threshold into the stroke-decision workflow could be beneficial for supporting neurointerventionalists in predicting underlying ICAD etiology or identifying “fast/slow stroke progressors". Hence, our findings are consistent with previously published data and HIR < 0.25 dichotomization could be considered for this purpose. Although other CTP automated parameters, such as T_max_ > 4 s/T_max_ > 6 s ≥ 2, have been proposed as potential markers for ICAS-LVO; these parameters remain non-validated and shown to be less determinant than HIR in predicting ICAS-LVO in our population^5^.

The development of a predictive tool with these patient characteristics could help in the early identification of patients who might benefit from a frontline EVT approach different than the usual stentretriever or direct aspiration techniques, considering treatments such as antiplatelet loading and balloon angioplasty + /− stenting at an early stage (either as a first technique or following a failed first-pass thrombectomy attempt). However, there is no robust evidence available to support an optimal treatment strategy for patients with ICAS-LVO undergoing EVT and prospective studies are needed. The ongoing Registry of Emergent Large veSsel oCclUsion duE to IntraCranial AtherosclerosiS (RESCUE-ICAS, ClinicalTrials.gov Identifier NCT05403593) and The Rescue Stenting for Failed Endovascular Thrombectomy in Acute Ischemic Stroke (ReSET, ClinicalTrials.gov Identifier NCT03993340) will provide an important insight to design randomized controlled trials in this patient population.

### Limitations

This study has some limitations due to its inherent retrospective, observational and single-center analysis. Main limitations include the lack of independent imaging core laboratory to assess ICAD diagnosis, and the self-reported eTICI evaluation. Patients with absence of recanalization (eTICI 0) were unable to establish underlying ICAD if alternative etiology was present^29^, nonetheless these patients exhibit occlusions with a higher risk of reperfusion failure and would also benefit from an intensive treatment involving mechanical or pharmacological rescue measures. Furthermore, these study findings are confirmed in the sensitivity analysis of patients with successful reperfusion.

Even though ICAD is the most common cause of stenosis/re-occlusion, in some cases it may indicate iatrogenic dissection or focal vasospasm. An artificial intelligence automated tool to detect IAC rapidly is warranted. Another limitation is the lack of a standardize decision tree on the adoption of rescue treatments, that were decided patient by patient according to the interventionalist criteria.

Although the model maintains an adequate predictive capability in our cohort, due to the potential limitations of the study, its predictive ability could be lower in other populations. Hence, the results need to be replicated in multicenter studies and in other to replicate our findings and establish its usefulness in routine clinical practice.

## Conclusions

A combination of baseline clinico-radiological characteristics assessed before EVT could help to identify anterior circulation ICAS-LVO in non-Asian populations. Imaging biomarkers such as intracranial artery calcifications, collateral status and occlusion location are associated with ICAS-LVO. This approach could be useful to perform a rapid assessment of the underlying etiology, thus guiding a patient-tailored thrombectomy. Prospective studies are needed to validate these novel findings in other populations.

### Supplementary Information

Supplementary Table 1.

## Abbreviations

AIS: Acute Ischemic Stroke
ICAD: Intracranial Atherosclerotic Disease
ICAS-LVO: Intracranial atherosclerosis related large vessel occlusion
EVT: Endovascular treatment
MT: Mechanical thrombectomy
CTP: Computed tomography perfusion
LVO: Large vessel occlusion
