# Supplementary material for: Clinico-radiological features of intracranial atherosclerosis-related large vessel occlusion prior to endovascular treatment
Source: Sci Rep. 2024 Feb 5;14:2945. doi: 10.1038/s41598-024-53354-z (PMC10844212; doi:10.1038/s41598-024-53354-z)
Supplement: Supplementary file 1 — Supplementary Table 1. [file 41598_2024_53354_MOESM1_ESM.docx]

**SUPPLEMENTAL MATERIAL**

|  | **Final diagnosis** | | |
| --- | --- | --- | --- |
|  | **ICAD**  **(n=28)** | **Non-ICAD**  **(n=310)** | **P value** |
| **Intravenous thrombolysis** [n, %] | 9 (32.1%) | 107 (34.5%) | 0.80 |
| **First endovascular techniche** [n, %]  Combined techniche  ADAPT  Stentriever  Primary angioplasty and/or stenting  Other | **23 (82.1%)**  **0 (0.0%)**  **1 (3.6%)**  **4 (14.3%)**  **0 (0.0%)** | **259 (83.5%)**  **31 (10.0%)**  **16 (5.2%)**  **1 (0.3%)**  **3 (1.0%)** | **<0.001** |
| **Number of Passes** | **2 (2-4)** | **2 (1-3)** | **0.004** |
| **First-pass effect** [n, %] | **5 (17.9%)** | **167 (54.0%)** | **<0.001** |
| **Succesful reperfusion before rescue treatments** [n, %] | **6 (21.4%)** | **278 (89.7%)** | **<0.001** |
| **Needed rescue treatments** [n, %] | **17 (60.7%)** | **5 (1.6%)** | **<0.001** |
| **Final succesful recanalization** [n, %] | **20 (71.4%)** | **283 (91.3%)** | **0.003** |
| **Symptomatic ICH** [n, %] | 4 (14.3%) | 22 (7.1%) | 0.254 |

**Table S1. Angiographic outcomes**

Footnote: ICAD, intracranial atherosclerotic disease; ADAPT, A Direct Aspiration First Pass Technique; First-pass effect, Succesful reperfusion (eTICI ≥ 2b) after one attempt/pass of a mechanical thrombectomy device.
